# Direct vision endoscopic retrograde appendicitis therapy in the treatment of appendicitis with appendicolith in young women

**DOI:** 10.1055/a-2304-8243

**Published:** 2024-05-07

**Authors:** Dezheng Lin, Yuping Su, Zehui Guo, Qinghua Zhong, Jiancong Hu, Mingli Su, Xuefeng Guo

**Affiliations:** 1 373651 Department of Endoscopic Surgery, Sun Yat-sen University Sixth Affiliated Hospital, Guangzhou, China; 2 373651 Guangdong Provincial Key Laboratory of Colorectal and Pelvic Floor Diseases, Sun Yat-sen University Sixth Affiliated Hospital, Guangzhou, China; 3 Biomedical Innovation Center, Sun Yat-Sen University Sixth Affiliated Hospital, Guangzhou, China; 4 Department of Endoscopy, Yuexi Hospital of the Sixth Affiliated Hospital, Xinyi, China

A 31-year old woman who was preparing for pregnancy sought medical attention due to recurrent right lower abdominal pain. She was diagnosed with acute appendicitis with appendicolith after an ultrasound examination. Unwilling to undergo appendectomy, she opted for endoscopic intervention and underwent direct vision endoscopic retrograde appendicitis therapy.

**Fig. 1:**
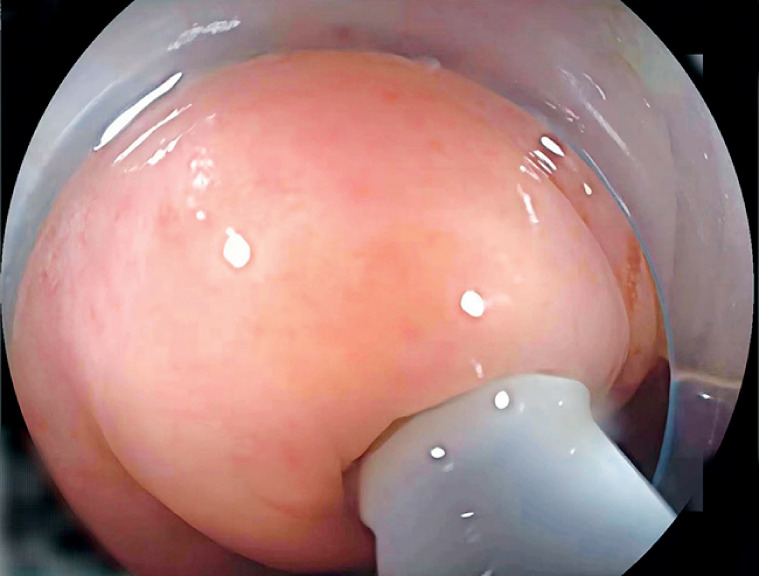
Appendiceal intubation.

**Fig. 2:**
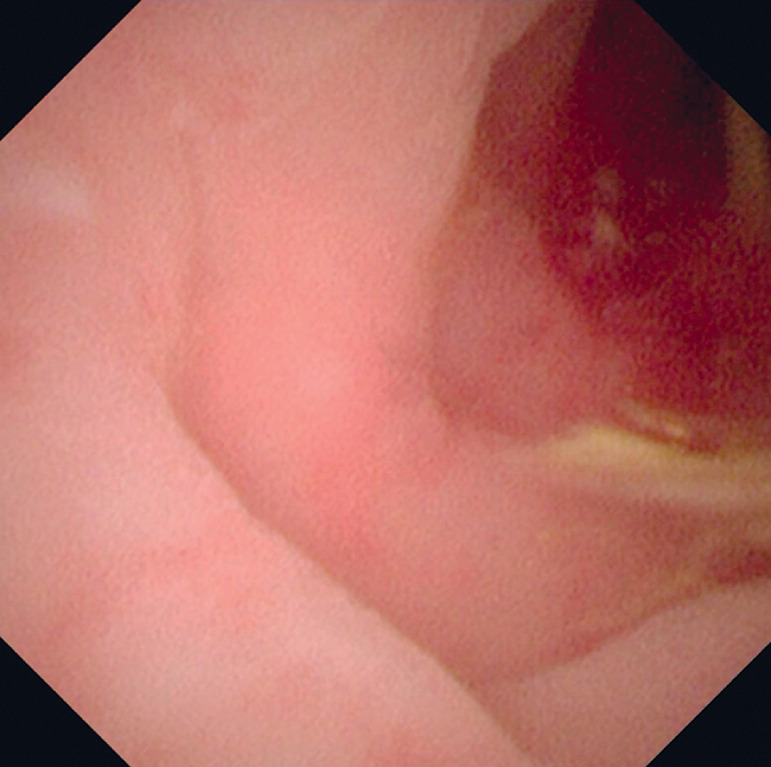
Direct vision showed congestion and swelling of the mucosa in the appendiceal cavity.

**Fig. 3:**
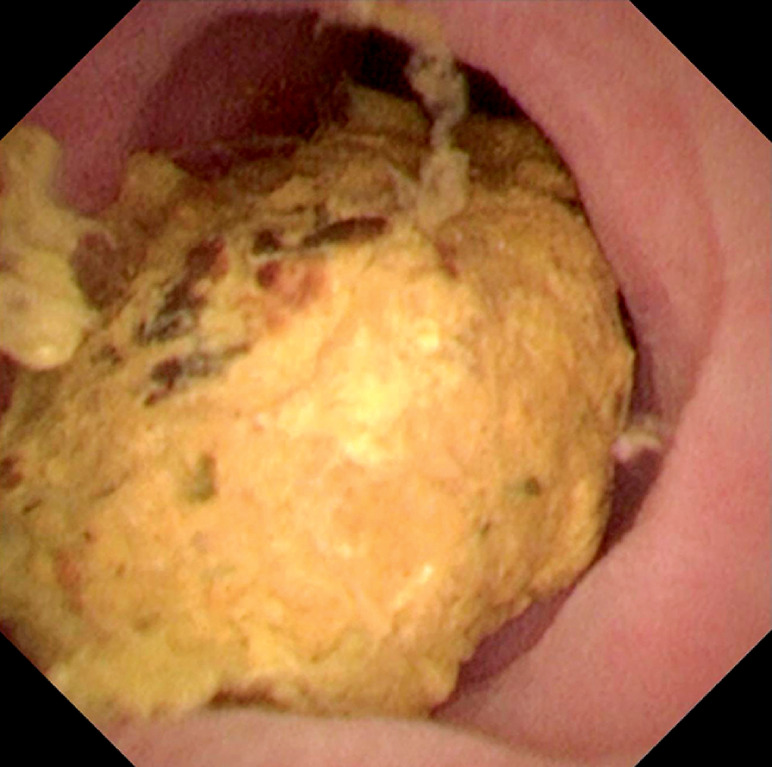
Appendicolith embedded in the middle of the appendiceal cavity.

**Fig. 4:**
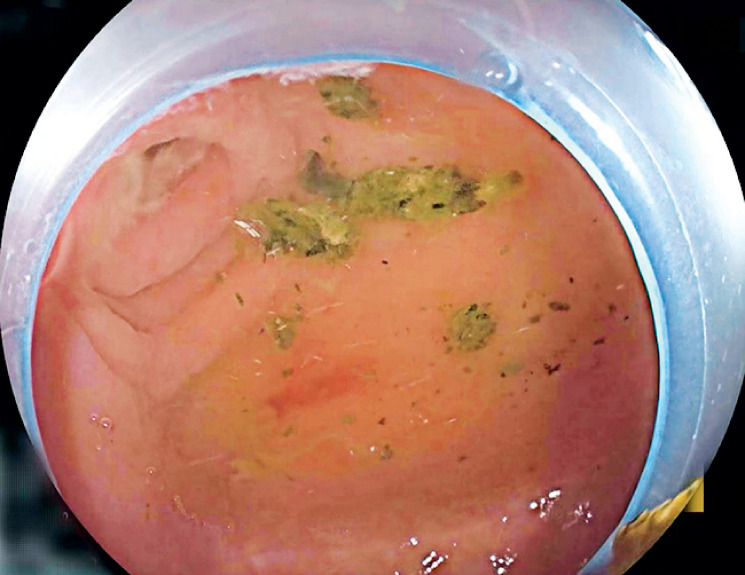
The removal of the appendicolith in cecum.

**Fig. 5:**
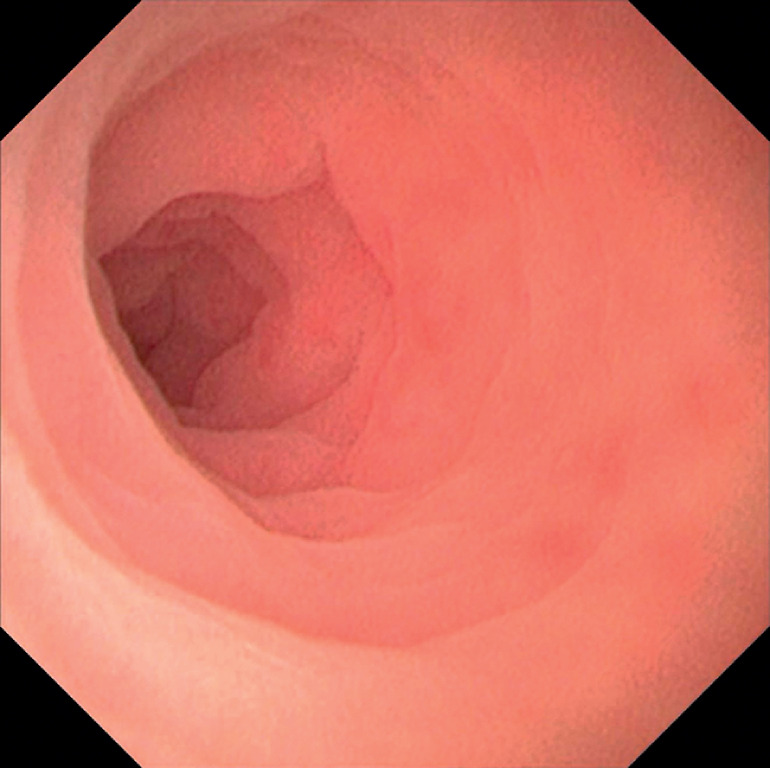
Subsequent reintubation and appendiceal irrigation.

The EyeMax subscope (Micro-Tech, Nanjing, China) was intubated into the appendiceal cavity, allowing direct observation of the mucosa ( Video 1 , Fig. 1 ). A metronidazole saline solution was used for irrigation of the appendiceal cavity. Subscope examination revealed congestion and swelling of the mucosa in the appendiceal orifice and cavity, confirming the diagnosis of acute appendicitis ( Fig. 2 ). An appendicolith, approximately 1 cm in size, was embedded in the middle of the appendiceal cavity and successfully removed using a basket ( Fig. 3 , Fig. 4 ). Subsequent reintubation of the subscope confirmed the absence of residual appendicolith ( Fig. 5 ). A 4-month follow-up showed no recurrence.

Direct vision endoscopic retrograde appendicitis therapy in the treatment of appendicitis with appendicolith in a young woman.Video 1

The EyeMax subscope is a direct vision system similar to Spyglass. The study emphasizes the utilization of the EyeMax subscope for direct visualization during observation, directed irrigation, and appendicolith removal. The absence of X-ray radiation makes it a suitable option for special populations, including children and pregnant women. Performing procedures under direct vision reduces the risk of incomplete flushing and stone removal, thereby lowering recurrence and misdiagnosis rates. Additionally, if needed, biopsy of appendiceal lesions can be conducted under direct vision.

Endoscopy_UCTN_Code_TTT_1AQ_2AI

